# PGC-1β modulates catabolism and fiber atrophy in the fasting-response of specific skeletal muscle beds

**DOI:** 10.1016/j.molmet.2022.101643

**Published:** 2022-11-16

**Authors:** Svenia Schmid, Barbara Heim-Kupr, Joaquín Pérez-Schindler, Shivani Mansingh, Markus Beer, Nitish Mittal, Nikolaus Ehrenfeuchter, Christoph Handschin

**Affiliations:** Biozentrum, University of Basel, Spitalstrasse 41, CH-4056 Basel, Switzerland

**Keywords:** Skeletal muscle, Fasting, PGC-1β, Myostatin, Atrophy, Ubiquitin proteasome

## Abstract

**Objective:**

Skeletal muscle is a pivotal organ for the coordination of systemic metabolism, constituting one of the largest storage site for glucose, lipids and amino acids. Tight temporal orchestration of protein breakdown in times of fasting has to be balanced with preservation of muscle mass and function. However, the molecular mechanisms that control the fasting response in muscle are poorly understood.

**Methods:**

We now have identified a role for the peroxisome proliferator-activated receptor γ coactivator 1β (PGC-1β) in the regulation of catabolic pathways in this context in muscle-specific loss-of-function mouse models.

**Results:**

Muscle-specific knockouts for PGC-1β experience mitigated muscle atrophy in fasting, linked to reduced expression of myostatin, atrogenes, activation of AMP-dependent protein kinase (AMPK) and other energy deprivation signaling pathways. At least in part, the muscle fasting response is modulated by a negative effect of PGC-1β on the nuclear factor of activated T-cells 1 (NFATC1).

**Conclusions:**

Collectively, these data highlight the complex regulation of muscle metabolism and reveal a new role for muscle PGC-1β in the control of proteostasis in fasting.

## Highlights

• PGC-1β transcription in muscle is controlled by the feeding state.
• Muscle-specific PGC-1β knockout mitigates fasting-induced muscle atrophy.
• Muscle PGC-1β affects different signaling pathways linked to ubiquitination and proteostasis.
• PGC-1β controls the balance between retaining muscle mass and catabolic processes.
• These findings are important to understand contexts of muscle wasting as well as protection, e.g. in hibernation.

## Abbreviations

AMPK: AMP-dependent protein kinase
ATP5A: ATP synthase 5 alpha
BSA: bovine serum albumin
β-OHB: β-hydroxybutyrate
BW: body weight
CaMK: Ca2+/calmodulin-dependent protein kinases
cAMP: cyclic AMP
CEE: chicken embryo extract
COX: cytochrome oxidase
Cox4i1: cytochrome C oxidase subunit 4 isoform 1
Cox5B: cytochrome C oxidase subunit 5B
CPT-cAMP: 8-(4-chlorophenylthio)adenosine 3′,5′-cyclic monophosphate
Creb: cAMP response binding protein
CSA: cross sectional area
CytC: cytochrome C
DEG: differentially expressed gene
DMSO: dimethyl sulfoxide
EDL: *Extensor digitorum longus* muscle
FC: fold change
FDR: false discovery rate
FoxO: forkhead box O
GO: gene ontology
HAS: human skeletal actin
IBMX: 3-isobutyl-1-methylxanthine
ISMARA: integrated motif activity response analysis
MAFbx: muscle atrophy F-box
minFeret: minimal fiber feret
MKO: PGC-1β-specific muscle knockout
Mstn: myostatin
MuRF-1: muscle RING finger 1
NDUFB8: NADH dehydrogenase 1 beta subcomplex subunit 8
NEFA: non-esterified fatty acid
Nfatc1: nuclear factor of activated T-cells, cytoplasmic 1
PGC-1: peroxisome proliferator-activated receptor γ coactivator-1
PKA: cyclic AMP-dependent protein kinase
RER: respiratory exchange ratio
SDH: succinate dehydrogenase
SDHB: mitochondrial succinate dehydrogenase iron-sulfur subunit
SEM: standard error of the means
TA: *Tibialis anterior* muscle
TBP: TATA-binding protein
TGF: transforming growth factor
UQCRC2: mitochondrial cytochrome b-c1 complex subunit 2
WT: wild type control

## 1 Introduction

Adequate partitioning of energy substrates in fasting and feeding is a fundamental process that involves a highly orchestrated and complex crosstalk of various organs [[1], [2], [3]]. Interplay between the brain, the liver, white and brown adipose tissue, the pancreas and the gastrointestinal tract determine appetite, satiety, the uptake, provisioning and distribution of substrates, as well as the balance between anabolism and catabolism. Skeletal muscle is centrally involved in this process, and, importantly, is the only organ that can be controlled in a conscious and voluntary manner to modulate energy metabolism [4,5]. The initial fasting response is dominated by a drop in blood glucose and the ensuing switch of pancreatic secretion from insulin to glucagon, triggering glycogenolysis and gluconeogenesis in the liver, and disrupting lipid deposition in adipose tissue as well as insulin-mediated glucose uptake into fat and muscle [6,7]. These early metabolic changes contribute to a stabilization of blood glucose levels, and thereby ensure subsistence to cells that are unable to use fatty acids as energy substrates such as red blood cells and the brain. Prolonged fasting, and the corresponding depletion of liver glycogen, leads to a more complete shift to hepatic, and to a lesser extent renal gluconeogenesis, fueled by glycerol from adipose tissue lipolysis and a subset of amino acids from skeletal muscle [[1], [2], [3]]. In addition, increased lipolysis in fat, and the production of ketone bodies help to support the energetic demands of various tissues, e.g. liver, cardiac and skeletal muscle. The production of glucogenic amino acids by proteolytic activity in muscle leads to a reduction of mass and function of this tissue. Therefore, a fine-tuning of skeletal muscle proteostasis is a central process in modulating systemic metabolism, tightly balanced between maintenance of muscular function and provisioning of amino acids for gluconeogenesis and other critical bodily functions [8]. After prolonged fasting and in starvation, muscle protective effects are superseded by the rising need for energy substrates, and to reduce energetically costly muscle tissue.

Many signaling pathways and transcriptional regulators have been proposed to be involved in the muscle fasting response, including the AMP-dependent protein kinase (AMPK), myostatin/activin receptors, nuclear factor κB (NFκB) and corticosteroid signaling [9]. Moreover, ubiquitin-proteasome activity and autophagy are central processes that promote protein degradation and amino acid liberation as energy or gluconeogenic substrates [10,11]. It however is unclear how these and potentially other signaling pathways are coordinated and integrated, and how the reported massive transcriptional change in the muscle fasting response is brought about [9]. In many tissues, the peroxisome proliferator-activated receptor γ coactivator 1 (PGC-1) proteins control cellular metabolic adaptation to various internal and external cues. For example, PGC-1α promotes thermogenesis in brown adipose tissue, gluconeogenesis in the liver, and adaptation of cardiac and skeletal muscle to changes in contractile activity [12]. Accordingly, PGC-1α transcription and posttranslational modifications of PGC-1α protein are activated by cold exposure, fasting and endurance exercise in these tissues, respectively. Hepatic PGC-1β mRNA levels are induced by fasting [13] and liver-specific PGC-1β knockout mice show a blunted fasting-refeeding response [14]. In skeletal muscle, gain-of-function of PGC-1β promotes a switch towards type IIx muscle fibers [15] and promotes a high-endurance phenotype [16], while loss of function studies provided evidence for a role in mitochondrial biogenesis and oxidative metabolism as well as antioxidant defense [17,18]. A potential regulatory and functional specification between PGC-1α and -1β could also be implied by the absence of cross-regulation in most contexts. Transgenic modulation of PGC-1α in skeletal muscle, either gain- or loss-of-function, did not affect gene expression of PGC-1β [19,20]. Similarly, muscle-specific ablation of the PGC-1β gene (PPARGC1B) did not alter PGC-1α transcription [18]. In contrast, muscle overexpression of PGC-1β, even though potentially at super-physiological levels, strongly reduced the transcript levels of PGC-1α [15]. Thus, in summary, in contrast to the robust regulation of PGC-1α gene expression by contractile activity and the ensuing effect on the exercise program, the regulation and function of PGC-1β in muscle are much less clear. Since feeding/fasting paradigms were shown to involve PGC-1β in the liver [13], the aim of our study was to elucidate the role of PGC-1β in the regulation of fasting-induced muscle remodeling.

## 2 Material and methods

### 2.1 Animal housing and PGC-1β muscle-specific knockout mouse generation

Mice had free access to food and water and were housed in a conventional facility with a 12 h light/12 h dark cycle. Experiments were performed with the approval of the Swiss authorities on adult male mice (10 weeks or older). PGC-1β muscle-specific knockout (MKO) animals were generated by crossing PGC-1β^loxP/loxP^ animals (Jackson Laboratory B6.129X1-Ppargc1b^tm1.1Dpk^/J, strain number 012378) [21] with human skeletal actin (HSA)-Cre transgenic mice. PGC-1β^loxP/loxP^ animals without Cre expression were used as wild-type control (WT) mice. Genotyping was performed from tail biopsies by PCR using specific primer pairs to detect the presence of the loxP sites, which resulted in amplicons of ∼500 bp (WT allele, 318 bp). Specific primer pairs to detect Cre recombinase resulted in amplicons of 100 bp in MKO mice. To confirm the knockout, 3–4 weeks old male and female mice were used. Male mice were examined in all other studies due to the higher muscle mass. Mice were randomized to fed and fasted groups.

### 2.2 In vivo analysis

To determine PGC-1 gene expression after maximal endurance performance, mice were acclimatized to treadmill running (Columbus Instruments) as described in Supp. Table 1 of the Supplemental Experimental Procedures. Two days after acclimatization, the test started at 5 m/min for 5 min and 8 m/min for 5 min with a 5° incline and the speed was increased 2 m/min every 15 min until exhaustion. 4 h after the exercise test, mice were killed by CO_2_ and *Quadriceps* muscles removed.

All mice were fed a normal chow diet. For fasting experiments, mice were placed in new, clean cages, and food was withdrawn from the mice in the morning. 24 h later, mice were killed by CO_2_ and organs removed.

Body composition of the mice was determined by qNMR using an EchoMRI-100™ analyzer (EchoMRI Medical Systems).

For indirect calorimetry, mice were placed in a CLAMS system (Columbus Instruments) for four days (2 days acclimatization, 2 days measurement) to assess their VO_2_ consumption, drinking behavior, food intake, locomotion and respiratory exchange ratio (RER). For body temperature measurements, anipills (Phymep S.a.r.l.; REF: 01101, LOT: 15-03,1 (DL 01-2017)) were implanted into the intraperitoneal cavity two weeks before CLAMS measurements.

### 2.3 Blood analysis

Blood analysis was carried out in the morning of *ad-libitum* fed or 24 h fasted animals. Blood glucose and ketone bodies were measured from tail blood with a glucose meter (Accu-Chek, Roche) or a ketone body meter (Precision Xtra, Abbott Laboratories), respectively. Plasma was obtained from whole blood, which was collected in microvette tubes (Sarstedt) and centrifuged at 2000 g for 10 min. Non-esterified fatty acids (NEFA) were measured in plasma using a NEFA-Kit (HR Series NEFA-HR (2), Wako Diagnostics) according to the manufacturer's instructions.

### 2.4 Primary cell culture

For the establishment of primary cell cultures, single fibers of 3 weeks old male WT mice were isolated as described in the Supplemental Experimental Procedures. Primary myoblasts were differentiated at around 60–70% confluency using differentiation medium (DMEM Glutamax, 4% HS, 1% P/S, 1% CEE) for 3 days. The next day, cells were serum starved with low glucose medium (LG, D6046, Sigma) for 16 h before treatment of the different compounds for 6 h. Compounds used were forskolin (100 μM, F3917, Sigma), 8-(4-chlorophenylthio)adenosine 3′,5′-cyclic monophosphate (CPT-cAMP, 100 μM, C3912, Sigma), 3-isobutyl-1-methylxanthine (IBMX, 1 mM, I5879, Sigma). All compounds were diluted in dimethyl sulfoxid (DMSO, 1%, D2650, Sigma).

### 2.5 Skeletal muscle stainings

Freshly isolated *Gastrocnemius* muscles were placed in 8% tragacanth (G1128, Sigma) and frozen in liquid nitrogen-cooled isopentane before cutting 10 μm cryo-cross-sections.

Succinate dehydrogenase (SDH) staining: sections were exposed to 50 mM sodium succinate (S2378, Sigma) in 0.1 M phosphate buffer in the presence of 0.5 mg/ml nitroblue tetrazolium (N5514, Sigma) for 30 min at 37 °C. Then sections were washed with ddH_2_O, dehydrated with ethanol and mounted with histomount (008030, Thermo Scientific).

Cytochrome oxidase (COX) staining: slides were exposed to 0.5 mg/ml 3,3′-Diaminobenzidine tetrahydrochloride hydrate (DAB, Sigma D5637), 0.2 mg/ml cytochrome c (C2506, Sigma) and 0.125 mg/ml catalase (C40, Sigma) in PBS for 1 h at 37 °C. Then slides were washed with ddH_2_O, dehydrated with ethanol and mounted with histomount (008030, Thermo Scientific).

Fiber typing was carried out as described in the Supplemental Experimental Procedures. Whole sections were pictured using a slide scanner (Axio Scan.Z1, Zeiss). For minFeret measurements and fiber typing counting, square pictures from total sections were cropped out (mean of two pictures in the oxidative part of the muscle, one in the glycolytic part of the muscle). For minFeret determination, a Fiji script was used as described in the Supplemental Experimental Procedures.

### 2.6 mRNA sequencing and analysis

mRNA sequencing library preparation was carried out as described in the Supplemental Experimental Procedures. Fastq files (GEO GSE210904) were mapped to the mouse genome (mm10) and RNAseq and statistical analysis performed with the CLC Genomics Workbench Software (Qiagen).

Differentially expressed genes were pictured in a Venn diagram with the use of the eulerAPE drawing tool [22]. Gene ontology (GO) analysis was executed by the usage of GeneCodis [23]. Only GO terms with at least 5 mapped genes were considered to be enriched and GO list was sorted according to corrected hypergeometric p-value. The top ten of enriched GO terms (Tables 3–7 in the Supplemental Experimental Procedures) were furthermore sorted by enrichment (calculated by: (Support/List Size)/(Reference Support/Reference size)). Annotation clusters were derived from DAVID [24] and heatmaps created with Morpheus (https://software.broadinstitute.org/morpheus). Integrated motif activity response analysis (ISMARA) was used to predict enriched transcription factor binding motifs [25].

### 2.7 RNA isolation and real-time qPCR

Total RNA was isolated from powdered *Quadriceps* and *Gastrocnemius* muscles or two pieces of liver with FastPrep tubes (MP Biomedicals) and TRI reagent (T9424, Sigma) according to the manufacturer's instructions. Total RNA from primary myotubes was isolated using the RNeasy Micro Kit (74004, Qiagen) according to the manufacturer's instructions (without DNase treatment). RNA concentration was measured with a NanoDrop OneC spectrophotometer (Thermo Scientific). RNA was treated with DNase I (18068015, Thermo Scientific) and then reverse transcribed using hexanucleotide mix (11277081001, Sigma) and SuperScript II reverse transcriptase (18064022, Thermo Scientific). The level of relative mRNA was quantified by real-time PCR on a StepOnePlus system (Applied Biosystems) using Fast SYBR green PCR master mix (4385612, Thermo Scientific) or on a Light Cycler 480 II system (Roche) using Fast Start Essential DNA Green Master mix (06924204001, Roche), respectively. The analysis of the mRNA was performed by the comparative ΔΔCT method using TATA binding protein (TBP) or 18S as endogenous controls as indicated in the figure legends. Primer sequences are listed in Supp. Table 2 of the Supplemental Experimental Procedures.

### 2.8 Protein isolation and Western blotting

Powdered tissue samples were homogenized with a polytron device in 300 μl of ice-cold lysis buffer (50 mM Tris–HCl, pH 7.5, 1 mM EDTA, 0.5 mM EGTA, 1% NP-40 substitute, 150 mM NaCl, 0.2% Na-deoxycholate, 1 mM DTT, fresh protease and phosphatase inhibitor cocktail, 10 mM nicotinamide). Samples were then shaken at 1300 rpm for 30 min at 4 °C, subsequently centrifuged at 13000 g for 10 min at 4 °C, and the protein concentration of the supernatant was determined by the Bradford assay (5000006, Bio-Rad). Equal aliquots of protein were boiled for 5 min in Laemmli sample buffer (250 mM Tris–HCl, pH 6.8, 8% SDS, 40% glycerol, 0.01% bromophenol blue, and 20% β-mercaptoethanol). Samples were separated on SDS-polyacrylamide gels and then transferred to nitrocellulose membranes. Membranes were blocked for 1 h in 5% bovine serum albumin (BSA) in Tris-buffered saline and Tween 20 (TBST) before overnight incubation at 4 °C with the appropriate primary antibody diluted in TBST (1:1000 dilution). Primary antibodies used are listed in the Supplemental Experimental Procedures. Following incubation, membranes were washed with TBS-T before incubation with an appropriate peroxidase-conjugated secondary antibody diluted in TBS-T (1:10′000 dilution). Antibody binding was detected using the enhanced chemiluminescence horseradish peroxidase (HRP) substrate detection kit (32106, Pierce). Quantification of Western blots was performed with the ImageJ software.

### 2.9 Glycogen isolation

Around 10 mg of powdered *Gastrocnemius* muscle were homogenized on ice in 200 μl water using a polytron device. Then, samples were boiled for 5 min in order to inactivate enzymes. After centrifugation for 5 min at 13,000 rpm, supernatant was moved to new tube and glycogen content measured using a glycogen assay kit (ab65620, Abcam) according to the manufacturer's instructions.

### 2.10 Generation of adenoviral vector

Adenovirus vectors were generated with the Adeno-X Adenoviral System 3 following manufacturer's instructions (Takara, #632267). Briefly, mouse PGC-1β was PCR-amplified from the plasmid (Addgene, #1031) plasmid. N-terminal HA and FLAG tags were introduced during PCR amplification, with the amplicon subcloned into the pAdenoX-ZsGreen1 vector to generate the HA-Flag-PGC-1 β adenovirus. Plasmid was corroborated via Sanger sequencing. Adenovirus was produced and amplified in Adeno-X™ 293 cells (Takara, # 632271), while titter was determined by fluorescence-activated cell sorting.

### 2.11 Adenovirus transduction

Cells were transduced with the HA-Flag-PGC-1β adenovirus at multiplicity of infection (MOI) 2. Adenovirus was prepared in the corresponding medium and cells were transduced for 4 h. Next, cells were washed once with phosphate buffered saline (PBS) and, then, incubated in adenovirus-free medium for a total of 48 h.

### 2.12 Forskolin stimulation

Forty-eight hours post-transduction with HA-Flag-PGC-1β adenovirus, cells were serum starved for 1 h and next incubated with DMSO alone (control) or 10 μM Forskolin (Sigma, #F3917) in DMSO for 6 h. Subsequently, cells were collected for immunoblot analysis as described, using the following antibodies: PGC-1β (Proteintech, #22378-I-AP), eEF2 (Cell Signaling Technology, #2332S), p-CREB^S133^ (Cell Signaling Technology, #9198S) and CREB (Cell Signaling Technology, #9197S).

### 2.13 Reporter gene assays

Reporter gene assays were performed in 96 well plates using 3× 104 HEK293 cells per well grown in growth medium without antibiotics. Cells were transfected using Opti-MEM™ (Thermo Fisher Scientific, #31985070) and polyethylenimine (Polysciences, # 23966). Plasmids and polyethylenimine were diluted in Opti-MEM™, following which they were mixed in a 1:3 ratio of μg DNA:μg polyethylenimine and incubated for 20 min at room temperature before adding to the cells. Cells were transfected 24 h after seeding with 5 ng pRL-SV40 (Promega, #E2231), 15 ng pNFAT-luc (Addgene, #17870), 10 ng pcDNA-NFAT, 40 ng pcDNA-f:PGC1b (Addgene, #1031). The total amount of plasmid DNA was kept constant at 110 ng per well by using the control plasmid pAdenoX-LacZ. Forty-eight hours after transfection, firefly and Renilla luciferase activities were measured with Dual-Glo® Luciferase Assay System (Promega, #E2920) following manufacturer's instructions. Renilla luciferase activity was used for normalization.

### 2.14 Statistical analysis

Values are expressed as means ± standard deviation (SD). Statistical significance was determined with unpaired two-tailed t-tests using Excel software and p < 0.05 was considered as significant. Significant differences between fed WT and fed MKO mice and fasted WT and fasted MKO mice, respectively, are indicated by an asterisk (∗). Significant differences between fed and fasted WT and fed and fasted MKO mice, respectively, are indicated by a hashtag (#).

## 3 Results

### 3.1 PGC-1β expression is downregulated in skeletal muscle by fasting

**Figure 1:**
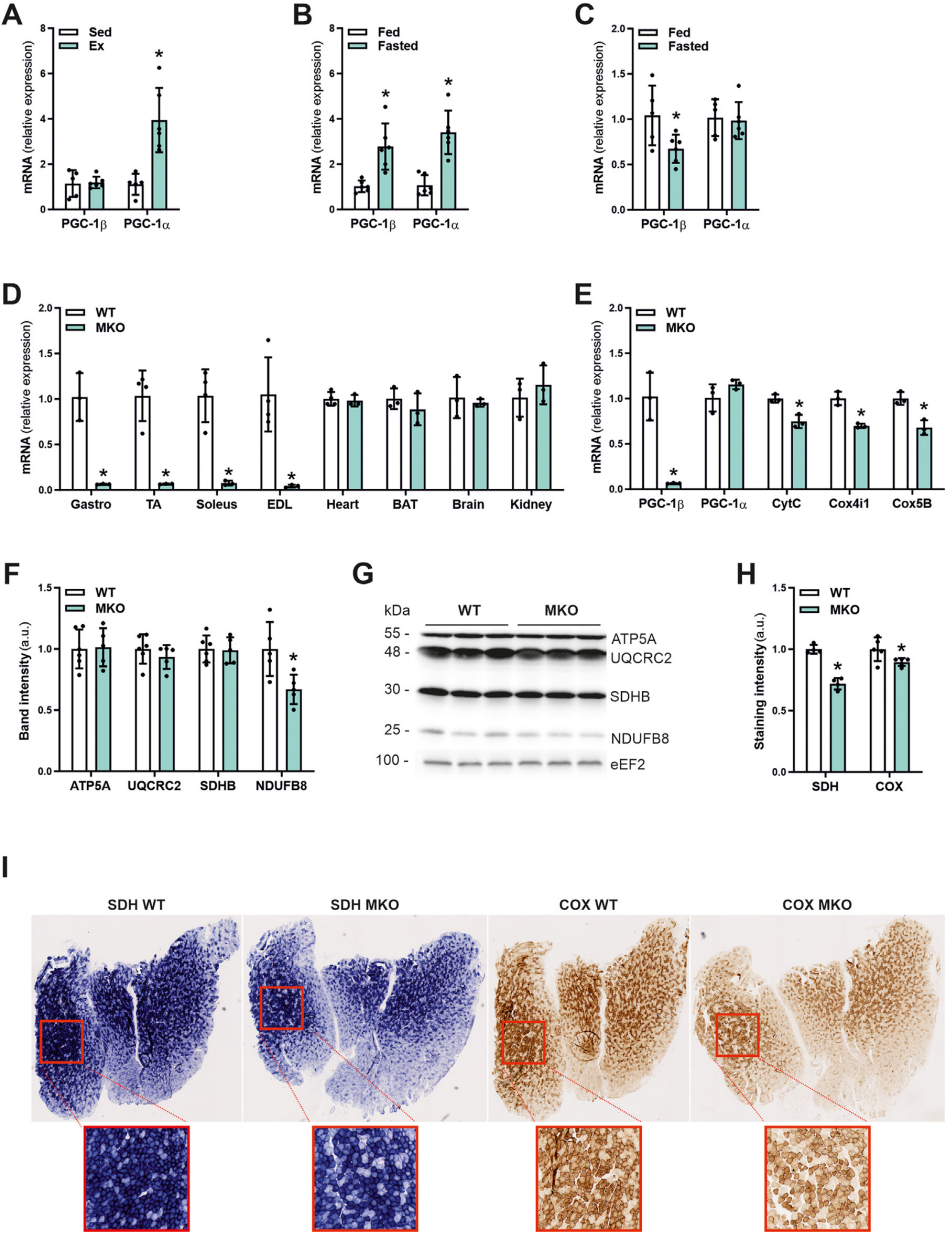
**PGC-1β expression is downregulated in skeletal muscle upon fasting.** (A) Gene expression of PGC-1β and PGC-1α relative to TATA-box binding protein (TBP) in *Quadriceps* muscle of sedentary or exercised mice. (B and C) Gene expression of PGC-1β and PGC-1α relative to 18S in liver (B) and *Gastrocnemius* muscle (C) of *ad-libitum* fed or 24 h fasted mice. (D) Gene expression of PGC-1β relative to TATA-box binding protein (TBP) in different muscles and other tissues of control (WT) and PGC-1β muscle-specific knockout (MKO) mice. (E) Gene expression of PGC-1β, mitochondrial target genes and PGC-1α relative to TATA-box binding protein (TBP) in *Gastrocnemius* muscle of WT and MKO mice. (F and G) Protein levels of different mitochondrial chain complexes (F) in *Gastrocnemius* muscle of WT and MKO mice and representative immunoblots (G). As a loading control eukaryotic elongation factor 2 (eEF2) was used. (H and I) Quantification of (H) and representative succinate dehydrogenase (SDH) and cytochrome oxidase (COX) stainings of *Gastrocnemius* muscle cryo-sections (I) of WT and MKO mice. ∗ indicates significant differences between sedentary and exercised mice, fed and fasted mice and WT and MKO mice; n = 3–6.

The regulation of PGC-1β in skeletal muscle is still largely unexplored, and might differ from that of PGC-1α. As a first step, we therefore interrogated the gene expression of PGC-1α and PGC-1β in exercise and fasting. In contrast to PGC-1α, PGC-1β transcript levels were unaffected by an acute bout of endurance exercise (Figure 1A), similar to previous observations [26,27]. Second, as published [28], both PGC-1α and PGC-1β transcripts were elevated by fasting in the liver (Figure 1B). Intriguingly, fasting resulted in a repression of PGC-1β in skeletal muscle, while PGC-1α transcription was unchanged (Figure 1C). Thus, these two factors differ in terms of regulation in skeletal muscle. PGC-1β furthermore exhibits diametrically opposite reactions to fasting in liver and muscle, indicative of a broader functional role for this coactivator in the fasting-feeding paradigm. To further explore this hypothesis, we generated PGC-1β MKOs by crossing HSA-Cre transgenic mice with floxed PGC-1β mice [21], leading to a deletion of exons 4-6 of the PGC-1β gene specifically in skeletal muscle while other tissues were unaffected (Figure 1D). Similar to other PGC-1β MKO models [18], no compensatory change in PGC-1α, but a broad effect on various mitochondrial target genes like cytochrome C (CytC), cytochrome C oxidase subunit 4 isoform 1 (Cox4i1) and cytochrome C oxidase subunit 5B (Cox5B) was observed under basal conditions (Figure 1E). PGC-1β ablation resulted in a reduction in the protein levels of only the mitochondrial NADH dehydrogenase 1 beta subcomplex subunit 8 (NDUFB8), part of complex I of the respiratory chain, while proteins belonging to other mitochondrial complexes, including ATP synthase 5 alpha (ATP5A), mitochondrial cytochrome b-c1 complex subunit 2 (UQCRC2) and mitochondrial succinate dehydrogenase iron-sulfur subunit (SDHB) were not altered by the ablation of Pppargc1b/PGC-1β (Figure 1F,G). Nevertheless, MKO mice showed a reduction in the enzymatic activities of the two mitochondrial enzyme complexes succinate dehydrogenase (SDH), entirely nuclear encoded, and cytochrome oxidase (COX), which consists of nuclear and mitochondrial encoded proteins (Figure 1H,I). A similar reduction in oxidative phosphorylation (OXPHOS) has been reported in other studies of skeletal muscle-specific loss-of-function of PGC-1β [17,18]. Taken together, our data validate this mouse model and revealed a hitherto undescribed regulation of PGC-1β in fasting in skeletal muscle.

### 3.2 PGC-1β MKO animals mitigate body weight loss after 24 h of fasting

**Figure 2:**
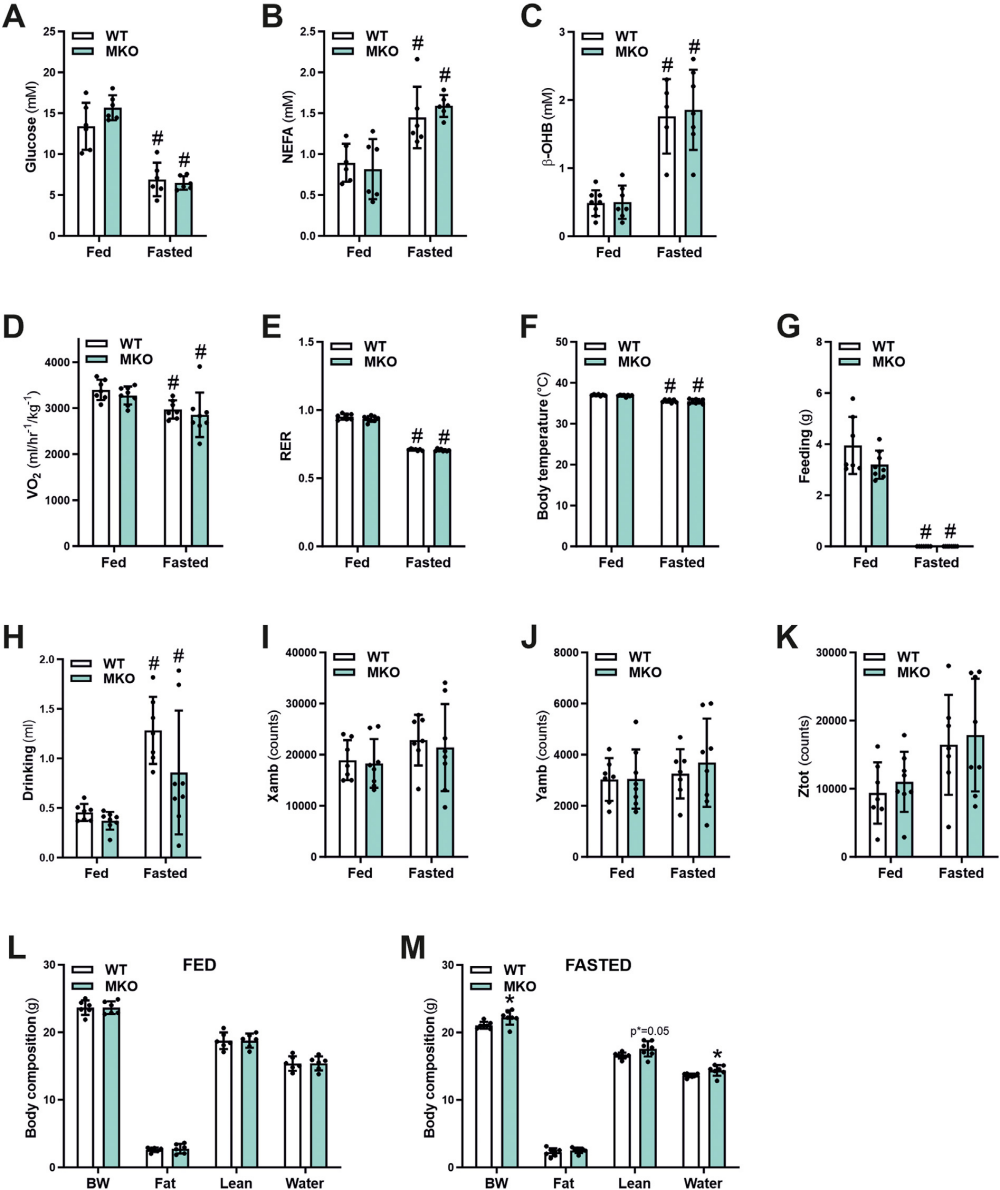
**MKO animals preserve body weight and lean mass after a 24h fasting period**. (A–C) Plasma glucose (A), non-esterified fatty acids (NEFA) (B) and β-hydroxybutyrate (β-OHB) (C) levels of *ad-libitum* fed or 24 h fasted mice. (D–K) Average oxygen consumption rate (VO_2_) normalized to body mass (D), respiratory exchange ratio (RER) (E), body temperature (F), food intake (G), drinking behavior (H), X- (I) and Y- (J) ambulatory activity and rearing activity (K) measured during nighttime by indirect calorimetry over a 48 h period in *ad-libitum* fed or 24 h fasted mice. L-M) Body composition measured by qNMR of *ad-libitum* fed or 24 h fasted mice with an initially identical body weight. ∗ indicates significant differences between WT and MKO mice; # indicates significant differences between fed and fasted conditions; n = 5–8.

To assess the consequences of the regulation of muscle PGC-1β in fasting, WT and MKO mice were food-deprived for 24 h. First, plasma glucose levels dropped after a 24 h fasting period, regardless of presence or absence of a functional PGC-1β in muscle (Figure 2A). Inversely, non-esterified fatty acids (NEFAs) (Figure 2B) and the ketone body β-hydroxybutyrate (β-OHB) (Figure 2C) plasma levels were significantly elevated after 24 h of fasting in WT and PGC-1β MKO animals. Similarly, assessment of the oxygen consumption rate (VO_2_) (Figure 2D), respiratory exchange ratio (RER) (Figure 2E), body temperature (Figure 2F), food consumption (Figure 2G) and drinking behavior (Figure 2H) under basal conditions revealed a significant effect of fasting on all of these parameters during nighttime, however again independent of the genotype. In addition, activity levels were similar between fed and fasted and WT and MKO animals (Figure 2I–K). In contrast, while body composition as measured by qNMR of *ad-libitum* fed mice was indistinguishable between the genotypes (Figure 2L), the MKO of PGC-1β mitigated the fasting-induced loss in body mass in comparison to their WT fasted counterparts (Figure 2M). Collectively, these results indicate that the systemic fasting response is not affected by muscle PGC-1β in a major way, albeit with a small, but significant effect on fasting-induced loss in body weight.

### 3.3 Muscle PGC-1β is necessary for fasting-induced fiber atrophy

**Figure 3:**
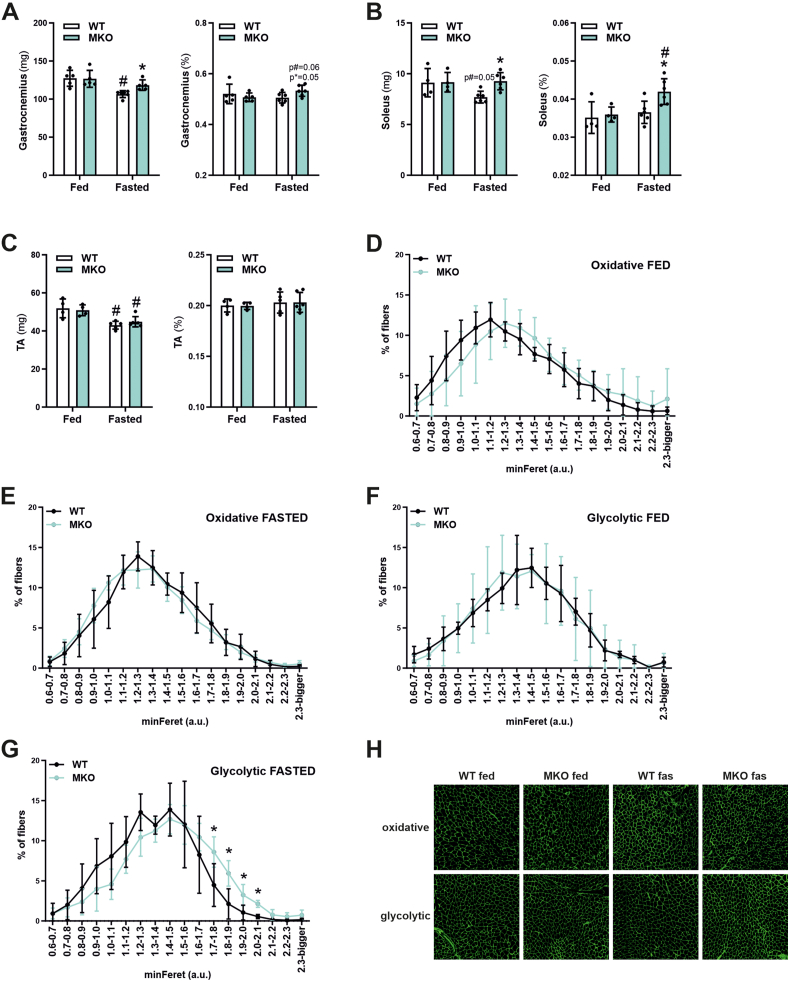
**PGC-1β is necessary for the fasting-induced fiber atrophy**. (A–C) *Gastrocnemius* (A), *Soleus* (B) and *Tibialis anterior* (TA) (C) absolute and relative muscle weights of *ad-libitum* fed or 24 h fasted mice. D-G) Minimal fiber ferrets (minFerret) of oxidative (D and E) and glycolytic (F and G) *Gastrocnemius* muscle cross-sections of *ad-libitum* fed (D and F) or 24 h fasted (E and G) mice. (H) Representative images of *Gastrocnemius* muscle cross-sections of *ad-libitum* fed or 24 h fasted WT and MKO mice. ∗ indicates significant differences between WT and MKO mice; # indicates significant differences between fed and fasted conditions; n = 4–6.

To assess the consequence of PGC-1β ablation on the fasting response in muscle, we measured individual muscle weights (absolute and relative) and fiber cross-sectional areas. *Gastrocnemius* (Figure 3A) and *Soleus* (Figure 3B), but not the *Tibialis anterior* (TA) (Figure 3C) muscles of fasted MKO animals were significantly heavier compared to fasted WT mice. To evaluate if the preserved muscle mass in fasted MKO animals is associated with the cross-sectional area (CSA) of single fibers, we analyzed the minimal fiber ferets (minFeret) in *Gastrocnemius* muscle cross-sections in two different areas, the center with a high proportion of oxidative, and the periphery with mostly glycolytic fibers. First, the CSA of oxidative fibers was not different between genotypes in fed (Figure 3D) and fasted (Figure 3E) conditions. Glycolytic fiber sizes were likewise indistinguishable between fed WT and MKO mice (Figure 3F). In contrast, bigger glycolytic fibers were found in fasted MKO compared to their WT counterparts (Figure 3G,H). Moreover, the fasting-induced reduction in glycolytic fibers in the WT animals was not observed in MKO mice (Supp. Figure S1A–F). For all the four groups, fiber type distribution was indistinguishable and thus cannot explain for the differences in CSA (Supp. Figure 1E). Together, these results demonstrate that at least in some muscles, and predominantly glycolytic fibers in which PGC-1β expression has been reported to be higher [15], fasting-induced loss in mass and fiber size depends on functional muscle PGC-1β.

### 3.4 Transcriptional changes induced by PGC-1β MKO are linked to muscle proteostasis

**Figure 4:**
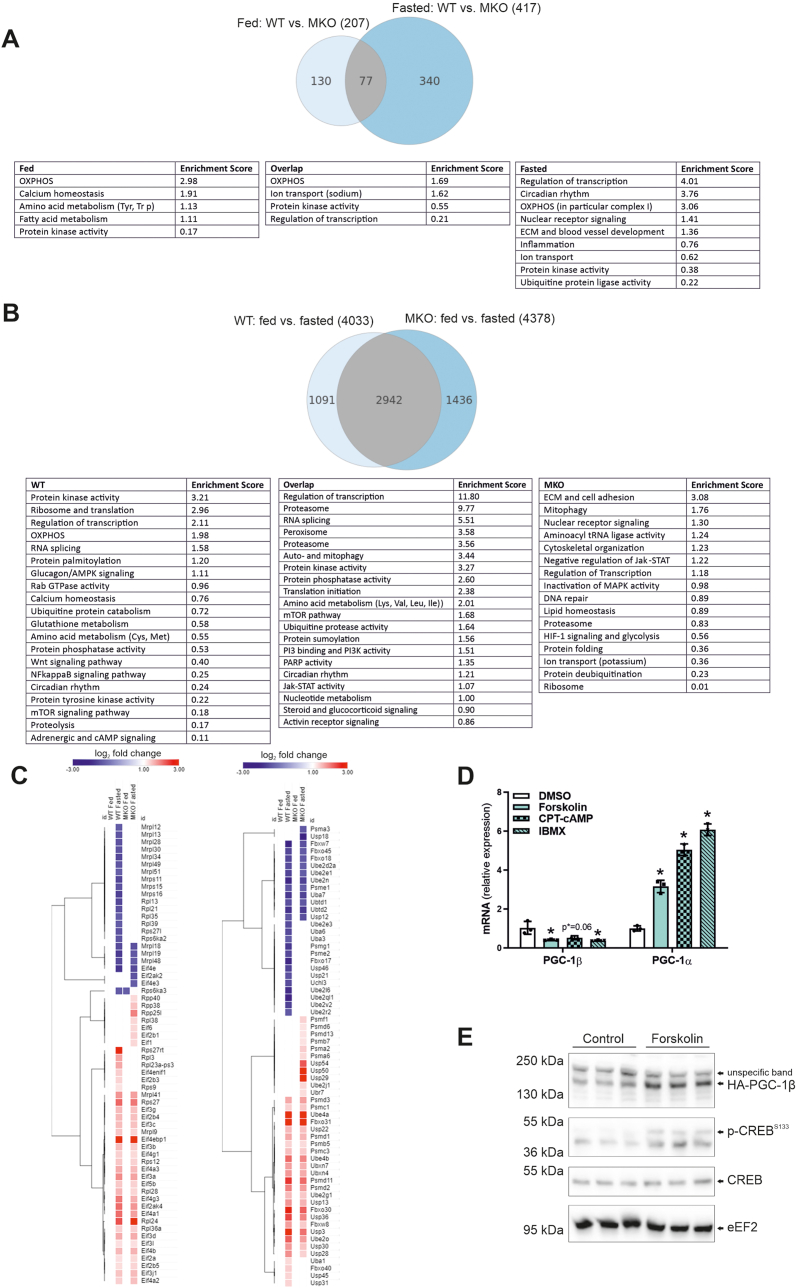
**Transcriptomic patterns in MKO link modulated muscle proteostasis to fiber atrophy**. (A) Gene expression changes in fed and fasted WT and MKO animals. A selection of Annotation Clusters indicates major predicted functional pathways that are altered only in the comparison of fed, in the overlap, and only in the comparison of fasted mice, respectively. (B) Gene expression comparison of the fasting response in WT and MKO mice. A selection of Annotation Clusters indicates major predicted functional pathways of physiological fasting that are dependent on muscle PGC-1β (WT only) and independent of muscle PGC-1β (overlap). The pathways found only in the comparison of MKO mice depict changes that arise de novo due to the absence of muscle PGC-1β in fasting. (C) Heatmaps of the relative expression of genes involved in protein synthesis and ribosome (left panel), and protein ubiquitination and proteasome (right panel). Colors represent the log_2_ fold change in gene expression compared to WT fed animals. (D) Gene expression of endogenous PGC-1β and PGC-1α relative to 18S in primary myotubes of WT mice treated with forskolin, 8-(4-chlorophenylthio)adenosine 3′,5′-cyclic monophosphate (CPT-cAMP) or 3-isobutyl-1-methylxanthine (IBMX) for 6 h (n = 3). (E) Protein levels of overexpressed, epitope-tagged (HA) PGC-1β, endogenous p-CREB^S133^, endogenous CREB and endogenous eEF2 in primary myotubes of WT mice treated with forskolin for 6 h (n = 3). (For interpretation of the references to color in this figure legend, the reader is referred to the Web version of this article.)

On a first glance, it seems curious that the main phenotypic differences between WT and MKOs emerge in the fasted muscle, in which endogenous PGC-1β gene expression is reduced, and not in fed animals in which PGC-1β transcript levels are higher (Figure 1C). To interrogate the mechanisms that could explain for this apparent discrepancy, transcriptomic analysis of fed and fasted WT and MKO *Gastrocnemius* muscles was performed. In line with the phenotypic observations of atrophy protection in fasting and minimal differences in the fed state, more genes were differentially expressed between fasted WT and MKO muscles (417 genes) compared with the corresponding fed muscles (207 genes), with only a small overlap of 77 genes between the two conditions (Figure 4A). A selection of Annotation clusters (the complete lists are attached as Supplemental Dataset 1) revealed that OXPHOS genes are different between WT and MKO across all comparisons (Figure 4A), with an enrichment of genes encoding for OXPHOS complex I primarily in the fasted condition. Notably, fasted MKO muscles exhibited a differential expression of genes related to ubiquitin protein ligase activity (Figure 4A). Second, the comparison of the physiological fasting response in WT animals to that observed in MKOs resulted in a split of around 27% (1091 out of 4033 genes) of gene expression changes that are dependent on muscle PGC-1β, and 73% (2942 out of 4033 genes) that respond to fasting in the presence or absence of muscle PGC-1β, at least in a qualitative manner (Figure 4B). Intriguingly, transcripts that are involved in proteostasis, e.g. those related to ribosome, translation, ubiquitin protein catabolism, NFκB and mTOR signaling, or proteolysis, were distributed over all three groups of genes (Figure 4B). These observations imply that different aspects of proteostasis are affected in these conditions. Indeed, the heatmaps depicting genes involved in ribosome and protein synthesis (Figure 4C, left panel), and in protein ubiquitination and proteasome (Figure 4C, right panel) underline a selective gene expression. For example, fasting regulation of a number of ribosomal proteins is strongly reduced in MKO muscle. Second, the control of the F-box protein 40 (Fbxo40) [29] and the ubiquitin-specific proteases 29, 50 and 54 (USP29/50/54) [30,31] in fasted WT and MKO muscles, respectively, indicate a potential shift in protein ubiquitination patterns elicited by PGC-1β towards deubiquitination in the MKOs, potentially linked to reduced atrophy. Together, these transcriptome analyses confirmed that, despite a downregulation of PGC-1β gene expression, the more extensive phenotypic difference between WT and MKOs occurs in the fasting context. It is conceivable that the ablation of muscle PGC-1β in the basal, fed-state, in which the difference between WT and MKO gene expression is larger, predisposes fasted muscles to react differently. It is also possible that gene expression levels might be misleading and not reflect PGC-1β protein content and/or activity. Unfortunately, the quantification of the levels of endogenous muscle PGC-1β by Western blots and mass spectroscopy was not possible, even when attempting immunoprecipitation before detection, and using synthetic peptides for targeted mass spectroscopy. Interestingly, a large overlap between the signaling pathways engaged in fasting and exercise exists, e.g. AMPK or protein kinase A (PKA)/cyclic adenosine monophosphate (cAMP) [32,33]. Thus, as an indirect proxy measure for PGC-1β protein levels, epitope-tagged, overexpressed PGC-1β was quantified after stimulation of muscle cells with pharmacological modulators of these fasting-associated signaling pathways. To assess the transcriptional and translational response, we therefore treated muscle cells with forskolin (a PKA activator), CPT-cAMP (a cAMP analog) and IBMX (a phosphodiesterase inhibitor). As expected, all three treatments promoted a robust induction of endogenous PGC-1α gene expression (Figure 4D). In contrast, transcript levels of endogenous muscle PGC-1β were reduced. These changes mimic the observed regulation of muscle PGC-1α in exercise (Figure 1A) and muscle PGC-1β in fasting (Figure 1C), respectively. In the same experimental paradigm, epitope-tagged, overexpressed PGC-1β was visualized by Western blot, and an increase in protein levels was detected (Figure 4E). Thus, these results imply that transcriptional regulation of PGC-1β could be dissociated from the corresponding protein content in fasted muscle, at least at certain time points, providing an explanation for the large transcriptional and phenotypic differences between WT and MKO mice in this setting.

### 3.5 Fasted MKO mice show a reduced induction of myostatin and atrophy marker gene expression

**Figure 5:**
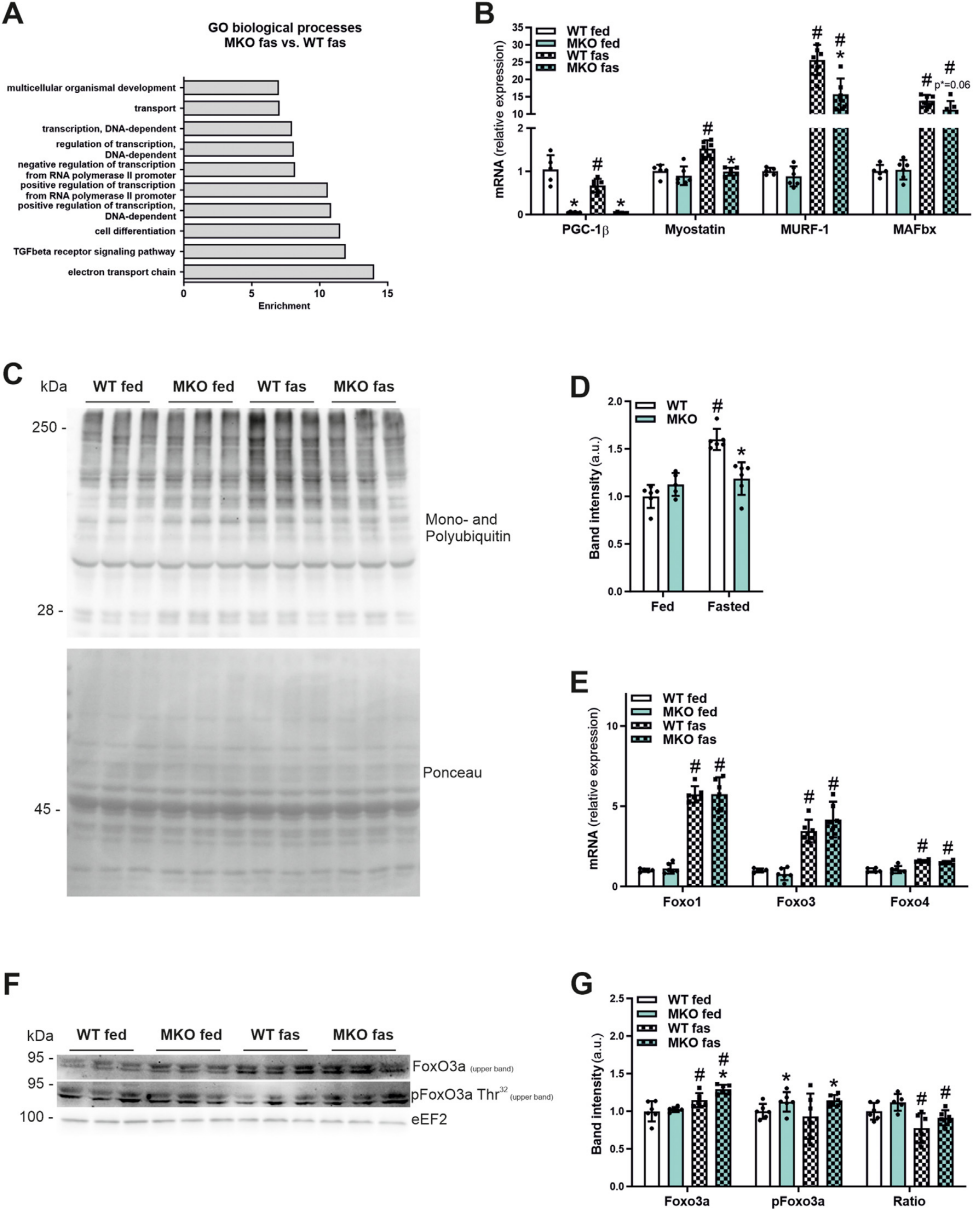
**Fasted MKO mice show reduced induction of myostatin, atrophy marker gene expression and protein ubiquitination**. (A) Gene ontology (GO) analysis of differentially expressed (DE) genes between fasted MKO vs. fasted WT mice. (B) Gene expression of PGC-1β, myostatin (Mstn), muscle RING finger 1 (MuRF-1) and muscle atrophy F-box (MAFbx) relative to 18S in *Gastrocnemius* muscle of *ad-libitum* fed or 24 h fasted mice (n = 5–6). (C and D) Representative immunoblot of ubiquitinylated proteins (C) in *Gastrocnemius* muscle of *ad-libitum* fed or 24 h fasted mice and corresponding quantification (D). Equal loading was verified by Ponceau staining of the membranes. (E) Gene expression of forkhead box O (Foxo) transcription factors 1, 3 and 4 relative to 18S in *Gastrocnemius* muscle of *ad-libitum* fed or 24 h fasted mice. (F and G) Representative immunoblots of total and phosphorylated forkhead box O 3a (Foxo3a) proteins in *Gastrocnemius* muscle of *ad-libitum* fed or 24 h fasted mice and corresponding quantifications (G). As a loading control eukaryotic elongation factor 2 (eEF2) was used. ∗ indicates significant differences between WT and MKO mice; # indicates significant differences between fed and fasted conditions; n = 3–6.

The differential analyses of gene expression between different conditions indicate a selective regulation of genes belonging to Annotation Clusters related to proteostasis in the presence or absence of muscle PGC-1β. Interestingly, in a more detailed interrogation, the top overrepresented GO categories in the comparison between fasted WT and MKOs included, besides “electron transport chain”, also “transforming growth factor (TGF)-β receptor signaling pathway” as one of the most significant terms (Figure 5A, Supp. Table 3). TGF-β signaling and in particular the associated differentially expressed gene (DEG) myostatin (Mstn) (Supp. Table 3) induce muscle atrophy by activating the ubiquitin proteolytic system [34,35]. Furthermore, Mstn is a well characterized negative regulator of skeletal muscle mass via small worm phenotype/mothers against decapentaplegic (SMAD) signaling [34]. In our RNAseq data, Mstn was significantly reduced in fasted MKO compared to fasted WT mice suggesting a downregulation of Mstn-associated TGF-β signaling. The RNAseq result was confirmed by qPCR revealing significantly lower Mstn expression in fasted MKO mice, and a fasting-linked induction that was confined to WT animals (Figure 5B). Moreover, the fasting induction of the ubiquitin ligase muscle RING finger 1 (MuRF-1) involved in muscle atrophy [35] was blunted in fasted MKO animals, in contrast to the muscle atrophy F-box (MAFbx) (Figure 5B). Functionally, the reduced expression of MuRF-1 is in line with with lower ubiquitination of proteins in fasted MKO animals, completely abrogating the increase in protein ubiquitination by fasting as observed in WTs (Figure 5C,D). Transcriptional induction of MuRF-1 is controlled by forkhead box O (FoxO) transcription factors [36], which include the three family members FoxO1, FoxO3, and FoxO4 [37]. In atrophic contexts, dephosphorylation of FoxO transcription factors leads to nuclear translocation and transcriptional induction of target genes [38]. The transcriptional regulation of all three FoxO members by fasting was similar between WT and MKO animals (Figure 5E). In contrast, phosphorylation of FoxO3 was elevated in fed and fasted MKOs, even though the increase in total protein in the fasted context was preserved (Figure 5F,G). These data suggest that the mitigated muscle atrophy might be associated with the blocked and blunted fasting induction of Mstn and MuRF-1 gene expression, respectively, as well as abrogated protein ubiquitination.

### 3.6 MKO mice show reduced activation of AMPK and PKA upon fasting

**Figure 6:**
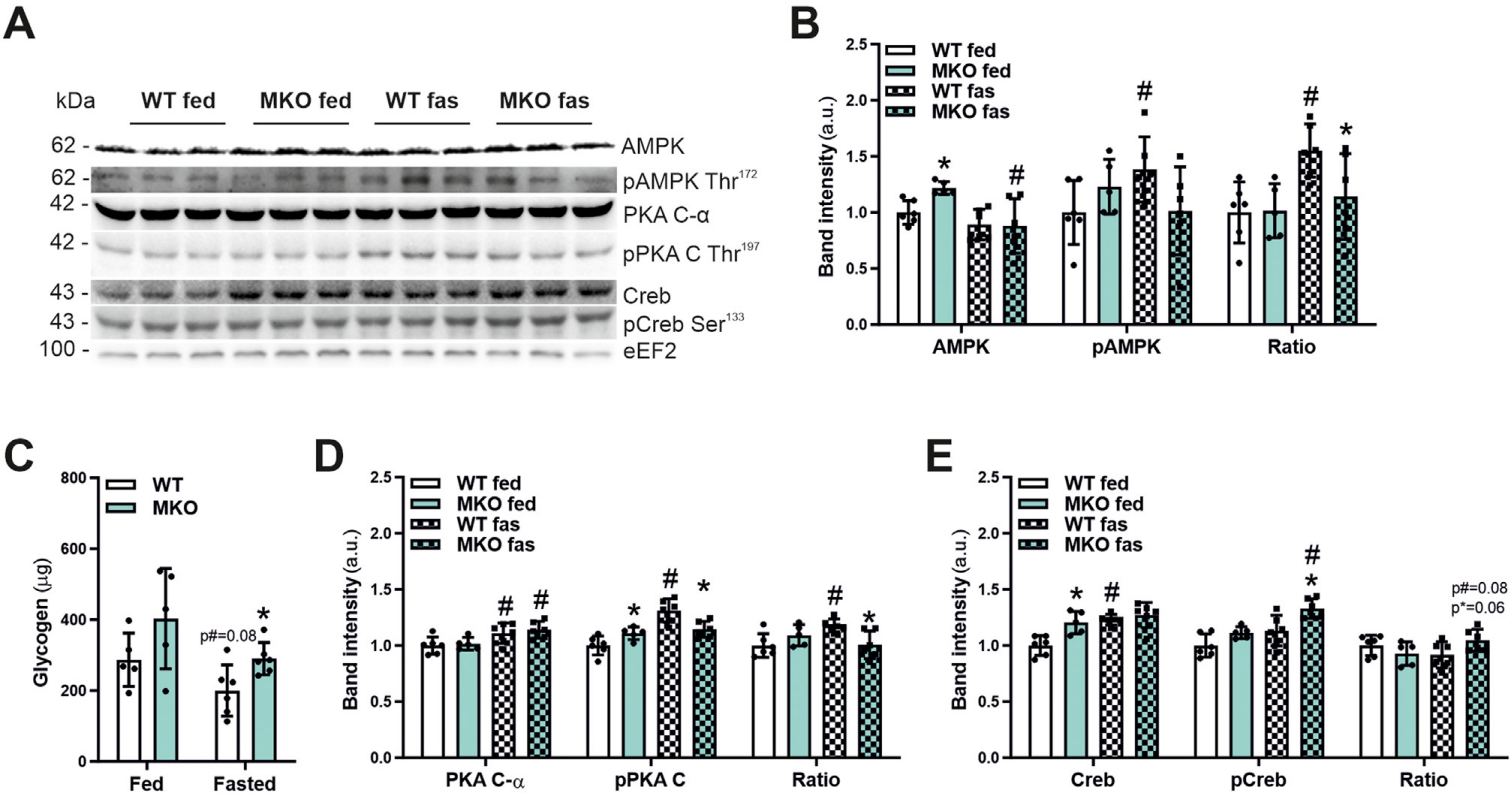
**MKO mice show reduced activation of AMPK and PKA in fasting.** (A, B and D, E) Representative immunoblots of total and phosphorylated AMP-dependent protein kinase (AMPK), cAMP-dependent protein kinase (PKA) and cAMP response element binding protein (Creb) protein levels (A) in *Gastrocnemius* muscle of *ad-libitum* fed or 24 h fasted mice and corresponding quantifications (B, D and E). As a loading control eukaryotic elongation factor 2 (eEF2) was used. (C) Total glycogen content in *Gastrocnemius* muscle of *ad-libitum* fed or 24 h fasted mice. ∗ indicates significant differences between WT and MKO mice; # indicates significant differences between fed and fasted conditions; n = 5–6.

To investigate further mechanisms by which muscle PGC-1β controls proteostasis, ubiquitination and fiber atrophy in fasting, we investigated major signaling pathways that are engaged in this context. AMPK is a central cellular energy sensor activated by changes in the intracellular AMP:ATP ratio. AMPK then promotes catabolic pathways in part to generate ATP, while concomitantly reducing anabolic pathways consuming ATP, respectively, e.g. by inhibiting the mammalian target of rapamycin (mTOR) and thereby protein biosynthesis [39]. Inversely to AMP, muscle glycogen directly binds to and inhibits AMPK in fed animals. In contrast to the short-term activation of AMPK in exercise leading to a metabolic remodeling and a training effect, more long-term activation of AMPK in fasting results in a catabolic state, in which protein synthesis is inhibited, and protein breakdown and autophagy are activated [5,39]. In fasting, elevated Mstn signaling further boosts AMPK signaling to regulate glucose and glycogen homeostasis in skeletal muscle [40]. Thus, we evaluated AMPK activity in fed and fasted WT and MKO animals. Correlating with lower Mstn expression, fasting-induced phosphorylation of AMPK as seen in WT was completely blunted in MKOs (Figure 6A,B). Moreover, AMPK activity could be additionally inhibited by higher muscle glycogen in fasted MKO compared to WT mice (Figure 6C). These findings could indicate that in the absence of muscle PGC-1β, fasting fails to adequately activate AMPK and thereby promote catalytic processes.

Intramuscular glycogen levels are regulated by various pathways and factors [41]. Catecholamine signaling, through activation of PKA, elicits strong glycogenolytic activity [42]. In light of the elevated fasting muscle glycogen levels in the MKOs (Figure 6C), we therefore interrogated PKA/cAMP signaling. Mirroring the activation pattern of AMPK (Figure 6A,B), PKA phosphorylation was elevated by fasting, but only in WT animals (Figure 6A,D). A cAMP-dependent downstream effector of the PKA signaling pathway, cAMP-response element binding protein (CREB) however exhibited a different pattern absent of fasting-linked changes in the ratio of phosphorylated to total protein, indicating that the phosphorylation of Serine 133 might be also affected by other kinases in this context (Figure 6A and E).

### 3.7 Nfatc1 activity is increased in fasted MKO animals

**Figure 7:**
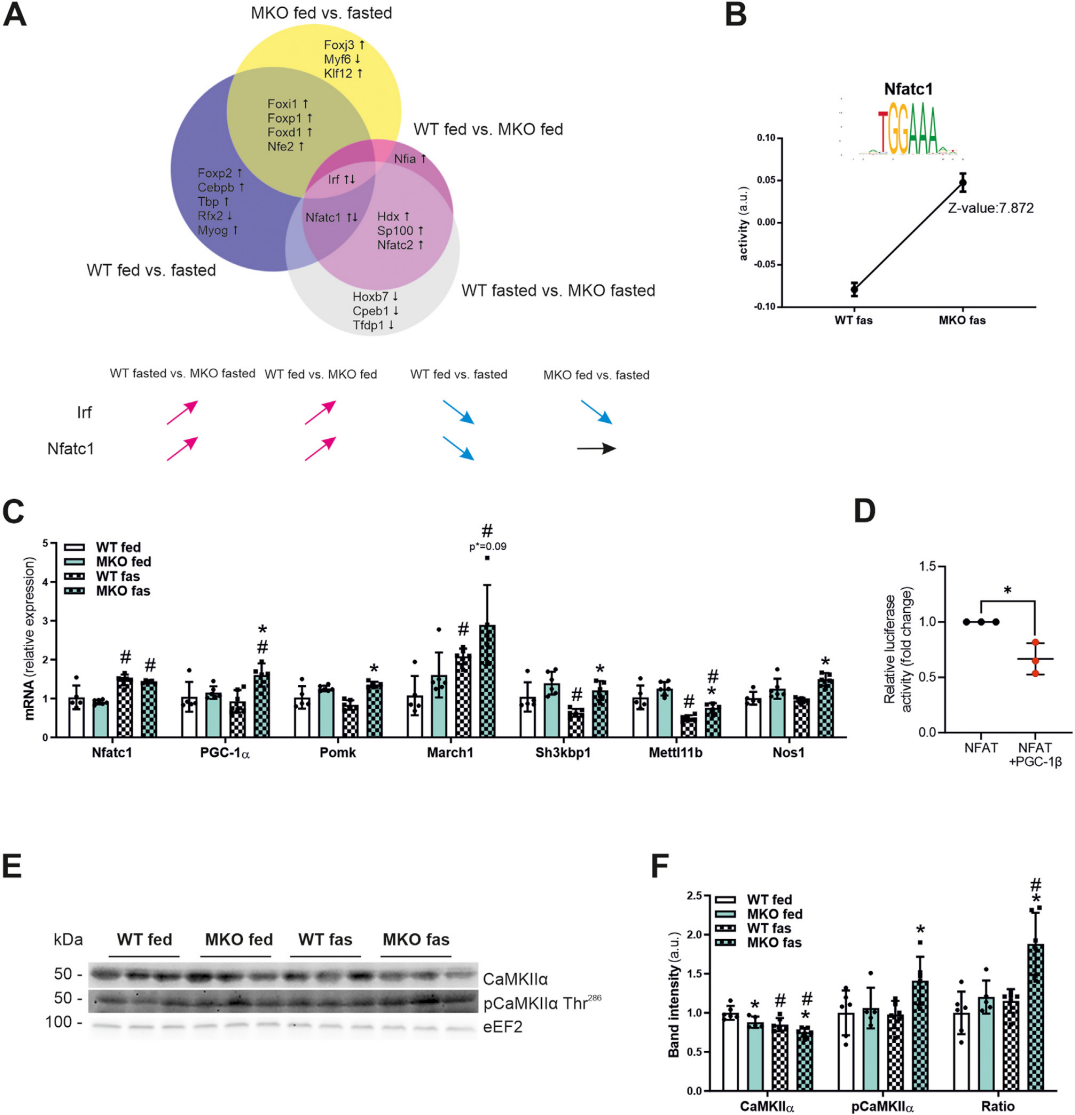
**Nfatc1 activity is increased in fasted MKO animals**. (A) ISMARA predictions of enriched transcription factor binding motifs in the comparison of fed and fasted WT and MKO mice, and the fasting response in WT and MKO animals. (B) Nuclear factor of activated T-cells, cytoplasmic 1 (Nfatc1) is the top ISMARA predicted motif from the gene expression profiles obtained in *Gastrocnemius* muscle of 24 h fasted WT and MKO mice. (C) Gene expression of Nfatc1 and predicted Nfatc1 target genes PGC-1α, protein-O-mannose kinase (Pomk), membrane associated ring-CH-type finger 1 (March1), SH3 domain containing kinase binding protein 1 (Sh3kbp1), methyltransferase like 11B (Mettl11b) and nitric oxide synthase 1 (Nos1) relative to 18S in *Gastrocnemius* muscle of *ad-libitum* fed or 24 h fasted mice. (D) Reporter gene assay using a 3× NFAT-luc plasmid, and co-transfection of NFATC1 alone or together with PGC-1β overexpression plasmids. (E and F) Representative immunoblots of total and phosphorylated Ca^2+^/calmodulin-dependent protein kinase IIα (CaMKIIα) protein levels in *Gastrocnemius* muscle of *ad-libitum* fed or 24 h fasted mice (E) and corresponding quantifications (F). As a loading control eukaryotic elongation factor 2 (eEF2) was used. ∗ indicates significant differences between WT and MKO mice; # indicates significant differences between fed and fasted conditions; n = 3–6.

To infer and predict potential transcription factors that are involved in the differential response of the MKO animals to fasting, we performed integrated motif activity response analysis (ISMARA) [25] on the RNAseq data. The activity of a number of transcription factor binding motifs were either associated with the genotype (presence or absence of muscle PGC-1β), or the feeding state (fasting response in WT or MKO *Gastrocnemius* muscles) (Figure 7A, Supplemental Dataset 2). Two motifs, Irf2_Irf1_Irf8_Irf9_Irf7 (abbreviated as Irf) and Nfatc1 were the only predicted binding sites with an interaction between genotype and feeding state (Figure 7A). Intriguingly, of those two, the fasting regulation of the Irf motif was independent of presence or absence of PGC-1β. However, the activity of the putative binding site for the nuclear factor of activated T-cells, cytoplasmic 1 (Nfatc1) was positively affected by the absence of PGC-1β, and was only reduced in activity in the WT animals upon fasting. In contrast, the MKO fasting response was devoid of a significant change in the Nfatc1 motif, implying that the repression of Nfatc1 in fasting might be dependent on PGC-1β. Nfatc1, a known target protein of the phosphatase calcineurin A [43], thus emerged as the most interesting candidate factor that could influence the fasting response in a PGC-1β-dependent manner. Accordingly, Nfatc1 activity was the top motif with highly elevated predicted activity in fasted MKO compared to fasted WT animals (Figure 7B). Interestingly, PGC-1α belongs to the predicted ISMARA Nfatc1 target genes. Others include protein-O-mannose kinase (Pomk), membrane associated ring-CH-type finger 1 (March1), SH3 domain containing kinase binding protein 1 (Sh3kbp1), methyltransferase like 11B (Mettl11b) and nitric oxide synthase 1 (Nos1). When assessing the transcriptional regulation of these Nfatc1 targets in fasted and fed WT and MKO muscles, all of these targets were elevated in fasted MKO animals except for March1, which did not reach statistical significance (Figure 7C). At least in part, the transcription of Nfatc1 targets was inhibited by fasting in WT animals (Figure 7C). Interestingly, Nfatc1 expression was significantly upregulated by fasting independently of the genotype (Figure 7C). These data imply a functional derepression of Nfatc1 in fasting in the absence of PGC-1β, and a potential repressive effect of elevated PGC-1β protein levels on this transcription factor activity in the physiological fasting context in the WT mice. We tested this hypothesis in reporter gene assays with Nfat response elements and indeed observed that cotransfection of PGC-1β reduced Nfatc1 activity (Figure 7D). The PGC-1 proteins have primarily been characterized as coactivators, and, at least in the case of PGC-1α, inhibition of gene expression is mediated in an indirect, secondary manner [44]. Since no transcriptional change in Nfatc1 expression by PGC-1β was found in the RNAseq data, we investigated upstream regulators of this transcription factor. The ratio of phosphorylated to total protein of the Ca^2+^/calmodulin-dependent protein kinases IIα (CaMKIIα) was greatly elevated in fasted MKOs (Figure 7E,F), which could be linked to the increased activity of Nfatc1 (Figure 7A) and phosphorylated CREB (Figure 6A,E) in this context.

## 4 Discussion

The PGC-1α and -1β coactivators are crucial regulatory factors in the control of cellular metabolism. A shared function of both proteins pertains to the activation of mitochondrial biogenesis and oxidative metabolism in different organs. PGC-1α exhibits a transcriptional regulation that is closely related to its function in tissue-specific adaptation to internal and external cues that elevate the energetic demand, e.g. cold exposure in brown adipose tissue or contraction in skeletal muscle. In contrast, the corresponding aspects of PGC-1β biology remain enigmatic, and this cofactor is understudied in terms of regulation, posttranslational modifications and biological function. In this manuscript, we report a strong inhibition of muscle PGC-1β by fasting, distinct from the regulation of PGC-1α in this context. Since PGC-1β levels are therefore higher in fed compared to fasted muscle, the observation of a more extensive phenotype in the fasted MKO was surprising. Similar, an at least partial protection of skeletal muscle against fasting-induced atrophy in the absence of PGC-1β was unexpected in light of previous results reporting a high endurance phenotype in the gain-of-function context [15,16], PGC-1β-mediated protection from denervation-induced disuse atrophy [45], stimulation of protein synthesis in muscle cells [46], and reduced antioxidant defense in the loss-of-function setting [18]. Our results thus demonstrate the very specific regulation of PGC-1α and PGC-1β in skeletal muscle, e.g. by exercise, fasting or cAMP signaling, linked to an overlapping yet also quite distinct functional outcome. Second, our data underline the large differences in PGC-1β-linked muscle atrophy mechanisms in different settings, e.g. denervation-induced disuse or fasting. Such a diversity was expected based on the narrow definition of atrogenes, genes that change in expression in different atrophy settings [36]. The common gene signature is primarily limited to the ubiquitin proteasome system, thus implying different upstream and downstream events as well as signaling pathways and molecular effectors to mediate muscle fiber atrophy in different physiological and pathological contexts.

In fasting, absence of a functional PGC-1β transcript mitigates muscle atrophy by a number of potential mechanisms. The modulation of proteostasis could be brought about by an altered expression profile of genes encoding proteins involved both in protein synthesis such a ribosomal proteins, including a cluster of mitochondrial ribosomal proteins, which are essential to balance hypertrophy and atrophy in muscle [47]. Second, the induction of MuRF-1 and Fbx proteins such as Fbxo40, which is also observed in denervation disuse atrophy [29,36], is absent in MKOs. Third, the prominent regulation of deubiquitinases [30,31] might contribute to the shift in overall protein ubiquitination in fasted MKO muscle. Next, knockout of muscle PGC-1β results in higher glycogen levels (that could conceivable contribute to the increased water content in these mice), and a lower activation of AMPK and other energy stress pathways, thereby alleviating pressure for initiation of protein degradation [39]. Then, loss-of-function of muscle PGC-1β mitigates fasting-induced elevation of Mstn, a strong pro-atrophic factor [34]. Moreover, PGC-1β represses the activity of Nfatc1, most likely by reducing the activity of upstream CaMK signaling. Nfatc1 contributes to insulin-like growth factor 1 (IGF-1)-activated hypertrophic signaling [48], and accordingly, elevated transcriptional function of Nfatc1 could help to reduce fiber atrophy in fasting in MKO mice. At least in part, the induction of the Nfatc1 target gene PGC-1α in the fasted MKO muscle also could contribute to atrophy mitigation [49]. Finally, the reduction in OXPHOS gene expression and mitochondrial activity in the MKOs might limit the supply of ATP for ubiquitin proteasomal activity. Of note, potential genotypic differences in coprophagic behavior was not assessed.

These results obtained in the loss-of-function setting collectively imply a strong catabolic role for muscle PGC-1β in fasting, linked to promoting protein degradation and amino acid liberation, fueled by mitochondrial oxidative metabolism of fatty acids and ketone bodies. Such a function however would have to be based on several premises: the transcriptional inhibition of muscle PGC-1β in the fasting context either indicates that the phenotype of the MKO is caused by a preconditioning in the fed state, or that opposite to the transcript levels, PGC-1β protein is stabilized and/or activated. Due to technical limitations to detect endogenous protein, we could only provide indirect proof for the latter scenario. Hopefully, future studies will enable the interrogation of the levels of endogenous muscle PGC-1β in fasting and feeding. Of note, since muscle overexpression of PGC-1β is not linked to muscle atrophy [15,16], posttranslational modifications and other mechanisms that specify the role of this coactivator in fasting might be expected. Overall, it is conceivable that the levels and function of PGC-1β, and thereby proteostasis in fasting, are tightly regulated to ensure an orchestrated balance between amino acid provisioning for gluconeogenesis and other critical functions, reduction of energetically costly muscle mass, vis-à-vis the necessity to safeguard muscle function for hunting and scavenging to bring fasting periods to an end. Therefore, stabilization and activation of the protein could be balanced by negative feedback loops on gene expression in the atrophic context. For example, the transcriptional repression of muscle PGC-1β by fasting is blunted in Foxo1 knockout mice [50].

In summary, our studies of PGC-1β MKOs revealed an unexpected regulation and function of this coactivator in fasting-induced muscle atrophy. Furthermore, these data also highlight the important role of PGC-1β in orchestrating a complex transcriptional network in a highly context-specific manner, affecting a number of different systems, e.g. myostatin, protein ubiquitination, ribosomal gene expression, glycogen depots, mitochondrial oxidative metabolism and several signaling pathways. Thereby, a coordinated response is elicited, confined and balanced by feedback mechanisms such as the transcriptional repression of the PGC-1β gene, at least in certain muscles, fibers and specific fasting time points. Whether the selective effects in muscles and fibers are due to differences in PGC-1β expression, motor unit engagement, muscle activity or other parameters is currently unclear. Nevertheless, these data indicate that this coactivator is important for the balance between preservation of muscle mass, and protein catabolism to liberate amino acids as energy substrates and to fuel hepatic gluconeogenesis, as well as to reduce the energetically costly maintenance of muscle mass. It is unclear whether the same mechanistic underpinnings of PGC-1β function are evolutionarily conserved, since at least some of the stress-related pathways that are engaged in the mouse are not affected by prolonged fasting in humans, at least at the conditions and muscles in which this was assessed [51,52]. Nevertheless, a better understanding of this biological program thus might help to address muscle atrophy contexts with overlapping etiologies and progression, for example cachexia, as well as muscle protective contexts such as hibernation.

## Data access

The transcriptomic RNAseq data have been deposited in GEO (accession number GSE210904). All other data are available from the corresponding author upon reasonable request.

## Author contributions

SS and CH conceived, designed and supervised the study. SS, BHK, JPS, SM, NB, NM and NE performed experiments. SS, BHK, JPS, SM, NM, NE and CH performed data analysis and interpretation. SS and CH wrote the manuscript. All authors reviewed and edited the manuscript, and approved the final version.

## Data Availability

Data will be made available on request.
