# Supplementary material for: PGC-1β modulates catabolism and fiber atrophy in the fasting-response of specific skeletal muscle beds
Source: Mol Metab. 2022 Nov 16;66:101643. doi: 10.1016/j.molmet.2022.101643 (PMC9723918; doi:10.1016/j.molmet.2022.101643)
Supplement: Multimedia component 1 [file mmc1.docx]

**Supplemental Material**

**Supplemental Experimental Procedures**

*Single fiber isolation and primary cell culture*

Before isolation of single fibers all dishes were coated with horse serum (HS, 16050122, Thermo Scientific) in order to prevent sticking of the fibers. Single fibers were isolated from *Extensor digitorum longus* (EDL) muscles of 3 weeks old male WT mice by microdissection. Muscles were digested in 0.2% collagenase A (10103586001, Roche) in DMEM Glutamax (31966021, Thermo Scientific) supplemented with 1% Penicillin/Streptomycin (P/S, 15140122, Thermo Scientific) for 1.5 h at 37 °C. Then EDLs were transferred to a new dish without collagenase for another 30 min. Single fibers were isolated by triturating the muscles with fire polished glass pipettes (pre-coated with HS) and distributed on matrigel coated plates (354234, BD Biosience). After incubation for 1 h at 37 °C fresh seeding medium was slowly added (DMEM Glutamax, 10% HS, 1% P/S, 0.5% chicken embryo extract (CEE, C3999, US Biological Life Sciences), 0.004% FGF-Basic Recombinant Human Protein (FGF, PHG0024, Thermo Scientific)).

After two days of incubation the medium was changed to proliferation medium (DMEM Glutamax, 20% FetalClone Serum (FCS, SH30066.03, GE Healthcare Life Sciences), 10% HS, 1% P/S, 1% CEE, 0.005% FGF). Two days after proliferation cells were washed with PBS and trypsinized (25300054, Thermo Scientific) for 5-10 min at 37°C. The reaction was stopped by the addition of HyClone medium (HyClone (SH30262.01, Thermo Scientific), 20% FetalClone Serum (FCS, SH30066.03, GE Healthcare Life Sciences), 10% HS, 1% P/S, 1% CEE, 1% L-glutamine (G7513, Sigma), 0.005% FGF). All cells were put on a non-coated dish for 1 h at 37 °C to let fibroblasts adhere. After incubation, floating primary myoblasts were carefully removed, counted and re-plated on new matrigel coated plates in HyClone medium.

*Fiber Typing and minFeret determination*

Sections were blocked in blocking solution (PBS + 0.4% Triton X-100 (93426, Sigma) + 10% goat serum (G9023, Sigma)) for 30 min. After washing with PBS sections were exposed to primary antibodies for 1 h at room temperature. Primary antibodies used were mouse IgG b2 MHC type1 (BA-F8, DSHB), mouse IgG 1 MHC type 2a (SC-71, DSHB) and laminin (ab11575, Abcam). After washing with PBS slides were incubated with the appropriate secondary antibodies AF647 IgG 2b goat-anti-mouse (A-21242, Life Technologies), AF568 IgG1 goat-anti-mouse (A-21124, Life Technologies) and AF488 IgG goat-anti-rabbit (A-11008, Life Technologies) for 1 h at room temperature. Finally, sections were washed with PBS, dehydrated with ethanol and mounted with ProLong Gold Antifade reagent (P36930, Life Technologies).

Fiji script for minFeret determination:

- run("3D Hysteresis Thresholding", "high=5 low=5");
- run("Analyze Particles...", "size=150-Infinity pixel circularity=0-1 show=Masks");
- run("Invert LUT");
- run("Dilate");
- run("Dilate");
- run("Dilate");
- run("Dilate");
- run("Skeletonize");
- run("Dilate");
- run("Invert LUT");
- run("Analyze Particles...", "size=500-Infinity pixel circularity=0.30-1.00 show=Overlay display exclude clear record add");
- drawAllFeretsDiameters();
- function drawAllFeretsDiameters() {for (i=0; i<nResults; i++) {x = getResult('XStart', i); y = getResult('YStart', i); doWand(x,y); drawFeretsDiameter(); if (i%5==0) showProgress(i/nResults); } run("Select None"); } function drawFeretsDiameter() {requires("1.29n"); run("Line Width...", "line=1"); diameter = 0.0; getSelectionCoordinates(xCoordinates, yCoordinates); n = xCoordinates.length; for (i=0; i<n; i++) {for (j=i; j<n; j++) {dx = xCoordinates[i] - xCoordinates[j]; dy = yCoordinates[i] - yCoordinates[j]; d = sqrt(dx*dx + dy*dy); if (d>diameter) {diameter = d; i1 = i; i2 = j; }}} setForegroundColor(255,0,0); drawLine(xCoordinates[i1], yCoordinates[i1],

xCoordinates[i2],yCoordinates[i2]);}

*mRNAseq library preparation and sequencing*

Polyadenylated mRNA was isolated from around 10 mg of powdered *Gastrocnemius* muscle using the dynabeads mRNA direct kit (61012, Thermo Scientific). Fragmentation of mRNA was carried out by alkaline hydrolysis (50 mM sodium carbonate pH 9.2, 1 mM EDTA) at 95 °C for 5 min. mRNA was cleaned and purified using the RNeasy MinElute Cleanup kit (74204, Qiagen). 3’- and 5’-ends were dephosphorylated by alkaline phosphatase treatment (FastAP, EF0651, Thermo Scientific) to prevent re-ligation of the mRNA fragments. Before mRNA was cleaned and purified as described above, 5’-OH-ends were re-phosphorylated by the usage of a T4 polynucleotide kinase (EK0032, Thermo Scientific) for 1 h at 37 °C. Addition of the 3’-adapter (5‘-(5rApp)-TGGAATTCTCGGGTGCCAAGG-(3SpC3)-3‘, 1 µmol, Integrated DNA Technologies) was carried out using a truncated and mutated T4 RNA ligase (M0351L, BioLabs) overnight at 4 °C. mRNA was cleaned and purified as described above before addition of the 5’-adapter (5’-GUUCAGAGUUCUACAGUCCGACGAUC, 1 µmol, Microsynth) by the action of a T4 RNA ligase (AM2141, Thermo Scientific) overnight at 4 °C. mRNA was cleaned and purified as described above before reverse transcription using a reverse transcription primer (5’-GCCTTGGCACCCGAGAATTCCA, 1 µmol, Microsynth) and SuperScript II reverse transcriptase (18064022, Thermo Scientific). cDNA was amplified and labelled by the addition of a 5’-RNA PCR primer (5’-AATGATACGGCGACCACCGAGATCTACACGTTCAGAGTTCTACA

GTCCGA, 1 µmol, Microsynth) and 3’-indexed RNA PCR primers (TruSeq Small RNA PCR primer sequences from Illumina, 0.2 µmol, Microsynth) using Taq DNA polymerase (D1806, Sigma). Finally, PCR product was purified by the Agencourt AMPure XP system (A63880, Beckman Coulter) and send for sequencing. Single read sequencing was performed with a GFB NextSeq 500 (R2D2) machine (81 cycles, Illumina RTA Version 2.4.6).

*Primary antibodies for Western blot analysis*

MitoProfile (ab110413, Mitosciences, Abcam), mono- and polyubiquitinylated conjugates monoclonal antibody (BML-PW8810, Enzo Life Sciences), FoxO3a (9467, Cell Signaling), p-FoxO3a (9464, Cell Signaling), AMPKα (2532, Cell Signaling), p-AMPKα (2531, Cell Signaling), PKA-Cα (4782, Cell Signaling), p-PKA C (4781, Cell Signaling), Creb (9104, Cell Signaling), p-Creb (9191, Cell Signaling), CaMKIIα (3362, Cell Signaling) and pCaMKIIα (12716, Cell Signaling). As a loading control eEF2 (2332, Cell Signaling) was used.

***Supp. Table 1. Acclimatization protocol for treadmill running at an incline of 5°.***

| Day 1 | 5 min  0 m/min | 5 min  5 m/min | 5 min  8 m/min | 10 min  10 m/min |  |  |
| --- | --- | --- | --- | --- | --- | --- |
| Day 2 | 5 min  0 m/min | 5 min  5 m/min | 5 min  8 m/min | 15 min  10 m/min | 5 min  12 m/min |  |
| Day 3 | 5 min  0 m/min | 5 min  5 m/min | 5 min  8 m/min | 15 min  10 m/min | 5 min  12 m/min | 2 min  14 m/min |
| Day 4 | 5 min  0 m/min | 5 min  5 m/min | 5 min  8 m/min | 15 min  10 m/min | 10 min  12 m/min | 2 min  14 m/min |
| Day 5 | 5 min  0 m/min | 5 min  5 m/min | 5 min  8 m/min | 15 min  10 m/min | 10 min  12 m/min | 5 min  14 m/min |

***Supp. Table 2. qPCR primer sequences***

| **Gene Name** | **Forward primer** | **Reverse primer** |
| --- | --- | --- |
| 18S | AGTCCCTGCCCTTTGTACACA | CGATCCGAGGGCCTCACTA |
| Cox4i1 | TACTTCGGTGTGCCTTCGA | TGACATGGGCCACATCAG |
| Cox5B | CTTCAGGCACCAAGGAAGAC | TTCACAGATGCAGCCCACTA |
| CytC | TGCCCAGTGCCACACTGT | CTGTCTTCCGCCCGAACA |
| FoxO1 | AATCCAGCATGAGCCCTTTG | CGTAACTTGATTTGCTGTCCTGAA |
| FoxO3 | CCGGACAAACGGCTCACT | GGCACACAGCGCACCAT |
| FoxO4 | CCACGAAGCAGTTCAAATGCT | TCAGACTCCGGCCTCATTG |
| MAFbx | CCAAAACTCAGTACTTCCATCAAG | CTATCAGCTCCAACAGCCTTAC |
| March1 | GGCCTACAACCGTGTGATCT | GCATCCTTGATTTCCGTGTT |
| Mettl11b | CCAGACAAGCGTCCTTTCTC | CAACGGGATCTAAAGGCAAA |
| Mstn | GCTGGCCCAGTGGATCTAAA | GCCCCTCTTTTTCCACATTTT |
| MuRF-1 | AGGCAGCCACCCGATGT | TCACACGTGAGACAGTAGATGTTGA |
| Nos1 | CAAGCCAAAGGGTAGCAGAG | ACCTCAGACCCAGCTCAAGA |
| PGC‐1α | TGATGTGAATGACTTGGATACAGACA | GCTCATTGTTGTACTGGTTGGATATG |
| PGC-1β | ATGCTTCCCTCACACCTCAG | GCTTTTGCCTTGTAGGCTTG |
| Pomk | CTCCAGAGGCCAGAAGACAC | CAGTGACCTGCTTGGTTCAA |
| Sh3kbp1 | AGCACAGAAGGAAAGCCAAA | AGCTCACGGACTTGCATCTT |
| TBP | TGCTGTTGGTGATTGTTGGT | CTGGCTTGTGTGGGAAAGAT |

***Supp. Table 3.*** ***Fasted MKO mice show reduced induction of myostatin and atrophy markers gene expression***

Gene ontology (GO) analysis of differentially expressed genes (DEG) between fasted MKO vs. fasted WT mice and associated genes (n=3-5).

**Inventory of Supplemental Data sets**

Supplemental Dataset 1: Annotation clusters of all comparisons

Supplemental Dataset 2: ISMARA analysis

**Supplementary Figures**


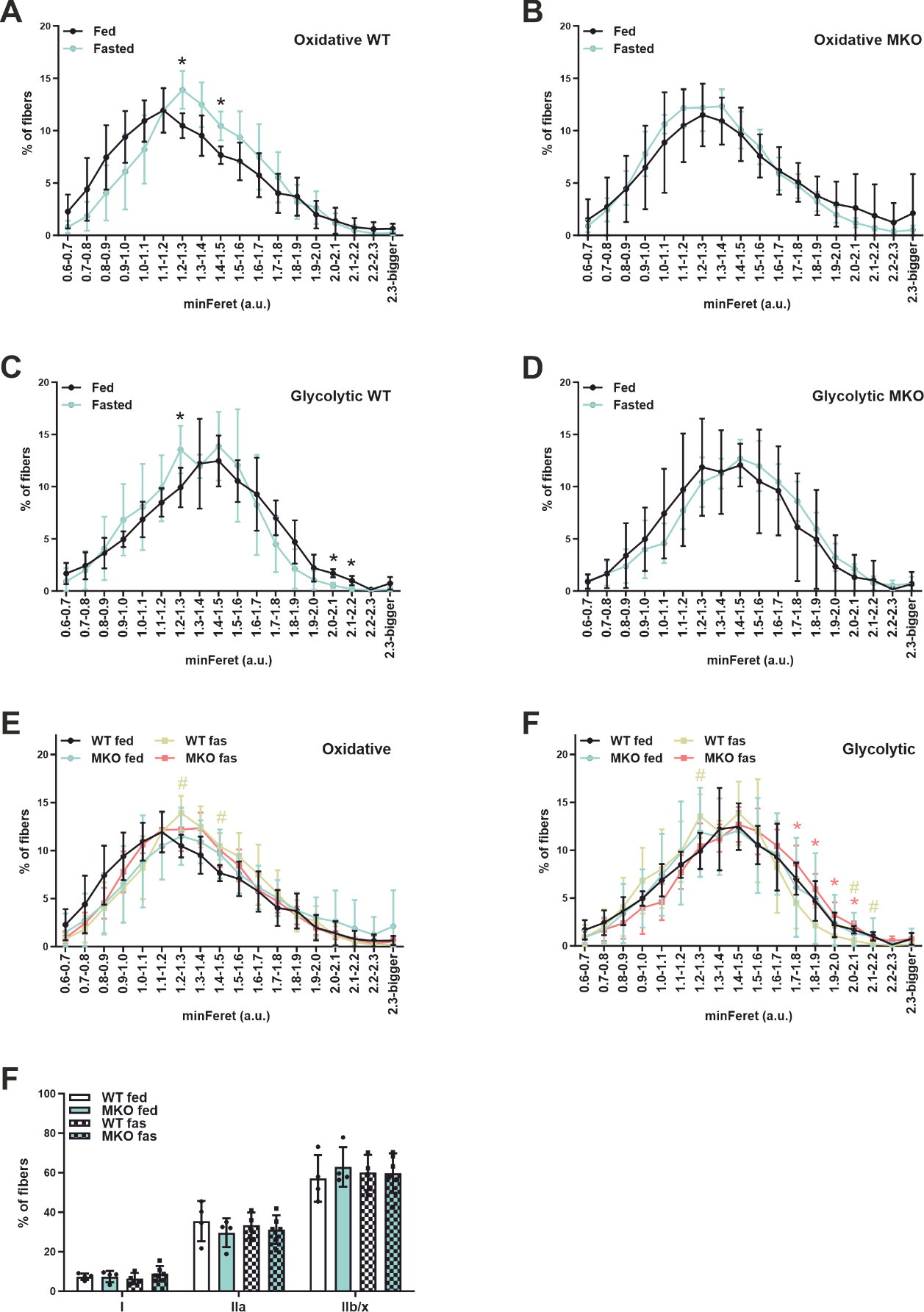


***Supp. Figure S1.*** ***PGC-1β is necessary for the fasting-induced fiber atrophy***

A-F) Minimal fiber ferrets (minFerret) of oxidative (A, B and E) and glycolytic (C, D aund F) *Gastrocnemius* muscle cross-sections of *ad-libitum* fed or 24 h fasted WT (A and C) and MKO (B and D) mice.

E) Quantification of type I, IIa and IIb/x fibers in *Gastrocnemius* muscle cross-sections of *ad-libitum* fed or 24 h fasted mice.

* indicates significant differences between WT and MKO mice; # indicates significant differences between fed and fasted conditions; color indicates comparison group; n=4-6.
